# Apple Polyphenols Decrease Atherosclerosis and Hepatic Steatosis in ApoE^−/−^ Mice through the ROS/MAPK/NF-κB Pathway

**DOI:** 10.3390/nu7085324

**Published:** 2015-08-24

**Authors:** Zhe-Rong Xu, Jin-You Li, Xin-Wei Dong, Zhong-Ju Tan, Wei-Zhen Wu, Qiang-Min Xie, Yun-Mei Yang

**Affiliations:** 1 Department of Geriatrics, First Affiliated Hospital, School of Medicine, Zhejiang University, Hangzhou 310003, China; E-Mails: xuzherong@yeah.net (Z.-R.X.); 20918253@zju.edu.cn (J.-Y.L.); yuanyuan17110@163.com (Z.-J.T.); yiyeqiu@126.com (W.-Z.W.); 2 Department of Pharmacology, School of Medicine, Zhejiang University, Hangzhou 310058, China; E-Mail: dxw826@zju.edu.cn; 3 Animal Laboratoy Center of Zhejiang University, Hangzhou 310058, China

**Keywords:** apple polyphenols, western-type diet, atherosclerosis, hepatic steatosis, atorvastatin, oxidative stress, vascular Inflammation, ApoE*^−/−^* mice

## Abstract

In this study, we examined the effects of apple polyphenols (APs) on hyperlipidemia, atherosclerosis, hepatic steatosis and endothelial function and investigated the potential mechanisms. ApoE^−/−^ mice were fed a western-type diet and orally treated with APs (100 mg/kg) or atorvastatin (10 mg/kg) for 12 weeks. Hyperlipidemia and atherosclerosis in the aortic sinuses and, and hepatic lipidosis were measured. The treatment with APs or atorvastatin induced a remarkable reduction in the atherosclerotic lesions and hepatic steatosis and decreased the levels of low-density lipoprotein, triglyceride, CCL-2 and VCAM-1 levels in the plasma. Conversely, the APs significantly increased the plasma levels of high-density lipoprotein (HDL) cholesterol and markedly up-regulated the glutathione peroxidase (GPx), catalase (CAT) and superoxide dismutase (SOD) levels in liver tissues. Moreover, the APs treatment modulated lipid metabolism by up-regulating the transcription of associated hepatic genes including *PPARα*, while down-regulating the transcription of *SCAP* and its downstream genes associated with lipid synthesis in the liver. Histological assessment showed that the APs treatment also reduced the macrophage infiltration in the aortic root plaque and the inflammatory cells infiltrations to the liver tissues. Moreover, we confirmed that the APs treatment greatly reduced the ox-LDL-induced endothelial dysfunction and monocyte adhesion to rat aortic endothelial cells (RAECs). Mechanistically, the APs treatment suppressed the ROS/MAPK/NF-κB signaling pathway, and consequently, reduced CCL-2, ICAM-1 and VCAM-1 expression. Our results suggest that the APs are a beneficial nutritional supplement for the attenuation of atherosclerosis.

## 1. Introduction

“An apple a day keeps the doctor away”. Epidemiological evidences support the idea that apple consumption is associated with a reduction in cardiovascular risk, partially due to the potent anti-oxidative effects of apple polyphenols (APs) [1]. For comparison, as comprising the most common group of plant polyphenols, the flavonoids exert potent anti-oxidant and anti-inflammatory effects [2,3,4]. The beneficial effects of flavonoids on atherosclerosis, including protection against the free radicals-induced tissue damage, have been proven in various medical and chemical studies [1,2,3,4,5]. The main active constituents of the APs include phloridzin, phlorizin, quercetin, catechins, procyanidins, epicatechin, rutin, and chlorogenic acid [5]. The APs are now receiving interest from scientists and consumers as dietary supplements due to their various beneficial effects on human health as effective dietary supplements [6]. More importantly, toxicological investigations have confirmed that the APs are safe and have little toxicity at average dietary level [7].

Both atherosclerosis and nonalcoholic steatohepatitis, which was formerly considered a lipid-storage disease, actually involves ongoing inflammatory responses. Inflammation in the arterial vessel wall is considered to play an important role in the pathogenesis of atherosclerosis. Indeed, recent advances have established a fundamental role for inflammation in mediating all stages of this disease, from initiation through progression [8,9,10]. The activation of monocytes and macrophages is an important initial step in the cascade of events that leads to many acute and chronic inflammatory diseases including atherosclerosis and nonalcoholic steatohepatitis. It is believed that the formation of foam cells, which are lipid-laden macrophages, is an early hallmark of atherosclerotic lesion formation. In humans, ongoing inflammatory reactions within the coronary atherosclerotic plaques are increasingly thought to be crucial determinants of the clinical course of patients with coronary artery diseases [11,12]. Likewise, in a various of animal models of atherosclerosis and nonalcoholic steatohepatitis, signs of inflammation are closely associated with the incipient lipid accumulation in the artery wall [13,14,15].

Atherosclerotic plaques tend to form particularly at the inner curvatures and branch points of arteries, regions that are often associated with disturbed blood flow. The formation is also enhanced by other factors such as a high plasma low-density lipoprotein (LDL) concentration [16]. The mechanical forces associated with blood flow have a profound effect on the properties of endothelial cells (ECs) of the arteries [16,17]. Thus, shear stress generally triggers an anti-atherogenic gene expression and signal transduction profile, which is lost at sites of disturbed blood flow [16,17]. In addition, sites with disturbed blood flow are associated with changes in the morphology of ECs, increases in the permeability to macromolecules such as LDL, and an accumulation of extracellular matrix (ECM) that causes the retention of particles such as LDL [17]. Cytokines can modulate EC permeability [16,17]. For instance, TNF-α cause reorganization of the actin and tubulin cytoskeletons in ECs, thereby opening up gaps between adjacent cells [18]. The pro-inflammatory chemokine, CCL-2 (monocyte chemoattractant protein-1, MCP-1), plays a fundamental role in monocyte recruitment and has been implicated as a contributing factor to atherosclerosis [18,19]. The activated ECs release a range of chemokines and other cytokines that then cause the recruitment of circulating immune cells, particularly monocytes and T lymphocytes [16]. In addition, the ECs express adhesion proteins, including intercellular adhesion molecule-1 (ICAM-1) and vascular cell adhesion molecule-1 (VCAM-1), which participate in the recruitment of the immune cells [16,17].

In this study, we investigated the modulating effects of the APs on the lipid levels in the plasma and the expression of hepatic antioxidant genes in a mouse model of hyperlipidemia. We also assessed the protective effect of the APs against the oxidative damage caused by the hyperlipidemia. Furthermore, we studied the influence of APs on the ox-LDL-induced ROS/MAPK/NF-κB activation and the subsequent expression of multiple cytokines and chemokines associated with the adhesion of monocytes. Our results may reveal the potential of the APs as a dietary supplement beneficial in preventing in the development of atherosclerosis.

## 2. Materials and Methods

### 2.1. Animal Experiments

The apple polyphenols (APs, purity > 75%) were a gift from JF-Natural (Tianjin, China). Atorvastatin (ATO) was purchased from Pfizer Ireland Pharmaceuticals (New York, USA). The experiments were approved by an independent Animal Care and Use Committee of Zhejiang University (Hangzhou, China). All animal experimental procedures were performed in accordance with the “Guide for the Care and Use of Laboratory Animals” and were in compliance with European Community specifications regarding the use of laboratory animals. Thirty 12-week-old male ApoE^−/−^ mice on C57BL/6J background were purchased from Beijing Vital River Laboratory Animal Technology and fed with western-type diet (21% fat from plant sources, 0.2% cholesterol) beginning when they were 12 weeks old. The mice were randomly divided into three groups: vehicle control group (WD, equivalent vehicle), atorvastatin group (ATO, 10 mg/kg/day) and APs group (APs, 100 mg/kg/day), in which the designated treatment was administered by the intragastric route. After 12 weeks of treatment, the mice were sacrificed, and the tissues were collected and immediately frozen.

### 2.2. Assessment of Aortic Atherosclerosis and Hepatic Histology

Sections of the aortic sinuses and the liver tissues were harvested, embedded in optimal cutting temperature compound (Sakura Finetek Europe B.V., Alphen aan den Rijn, The Netherlands) and then frozen. The frozen tissues were cut into 5-µm-thick cryosections and stained with 0.5% Oil Red O (Sigma-Aldrich, St. Louis, MO, USA) and hematoxylin. Quantification of the percentage of aortic atheroma was performed with computer-assisted planimetry of the oil-red O using Image Pro plus software as described [20]. Liver tissues that had been fixed in 10% formalin overnight were dehydrated in ethanol and finally embedded in paraffin. Paraffin sections were then sliced into 6 μm thick were cut and stained with hematoxylin and eosin. The infiltration of inflammatory cells in ten microscopic fields was counted at a 400× magnification. The macrophages content of the atherosclerotic plaque was visualized using immunostaining for CD68. Immunohistochemistry was carried out according to guidelines provided along with the Strept Avidin-Biotin Complex kit, with a 1:100 dilution of anti-CC68 antibody (both from Boster Bio-engineering Ltd. Co., Wuhan, China). The liver tissues were also used for the measurement of oxidative activity and quantitative real-time polymerase chain reaction (qRT-PCR) for the associated hepatic lipogenic gene.

### 2.3. Assessment of Hepatic Redox Status

The level of malondialdehyde (MDA) was assayed by monitoring the formation of thiobarbituric acid reactive substance formation. The enzymatic activities of CAT, GSH-Px and SOD were measured using commercial kits according to the manufacturer’s instructions (Nanjing Jiancheng Institute of Biotechnology, Nanjing, China).

### 2.4. Measurement of Body Weight, Hepatic Lipids and Metabolic Parameters

The body weight of each mouse was measured and recorded weekly beginning at 12 weeks of age. On weeks of 8 and 12, blood samples were collected from the lateral tail veins of the mice. The total hepatic lipids were extracted from the left lobe of each liver using a modified Folch method. The plasma TC and TG, as well as hepatic levels of TC and TG, were measured using a colorimetric enzymatic kit from Nanjing Jiancheng Institute of Biotechnology (Nanjing, China) according to Richmond’s colorimetric procedure and Trinder’s colorimetric method. The plasma levels of HDL-c and LDL-c were determined using enzymatic kits (Nanjing Jiancheng Bioengineering Institute, Nanjing, China). The plasma leptin and adiponectin levels were measured using enzyme-linked immunosorbent assay kits (Boster, Wuhan, China) according to the manufacturer’s instructions. The assays were read using a SpectraMax M2 Microplate Reader (Molecular Devices, Sunnyvale, CA, USA).

### 2.5. Cell Isolation and Adhesion Assay

Rat aortic endothelial cells were isolated from male Sprague-Dawley rats (150–180 g) by an explant method and cultured in RPMI-1640 growth medium (HyClone, Logan, UT, USA) supplemented with 10% fetal bovine serum, heparin, 100 U/mL penicillin, and 100 U/mL streptomycin at 37 °C in 5% CO_2_ atmosphere. When a significant and consistent outgrowth of endothelial cells occurred after 5–8 days in culture, the tissue pieces were removed to avoid contamination with fibroblasts. For the experiments, the rat aortic endothelial cells (RAECs) were used for three passages (number of times subcultured) and each assay was replicated three times in triplicate using passages 3–8. The RAECs were fixed with fresh 4% paraformaldehyde and immunostained for factor VIII and the nuclei were stained with DAPI. Images were obtained on a Zeiss Axio scope with the Axiovision software (Zeiss, Thornwood, NY, USA). Rat peripheral blood mononuclear cells (RPBMNCs) were isolated from the blood of rat by density gradient centrifugation with Ficoll. Then, the cells were resuspended in a serum-free medium at a density of 1 × 10^5^ cells/mL. RAECs were seeded in 12-well plates to reach confluent monolayers and pretreated with the APs (1–100 μg/mL for 1 h), then activated with 100 μg/mL ox-LDL for 3 h. The RPBMNCs were added to the activated endothelial cells and incubated for 3 h. The unbound RPBMNCs were then gently washed away with PBS. The monocyte adhesion was quantified by counting the cells using a phase-contrast microscope.

### 2.6. Cell Viability Assay and ROS Detection

The RAECs were plated into a 96-well plate and stimulated with different concentration of APs (at 1 μg/mL, 10 μg/mL and 100 μg/mL). After 24 h, the cells were washed with PBS 3 times with PBS, supplied with 0.1 mL fresh media containing 0.5% MTT (MTT Sigma, St. Louis, MO, USA) and incubated at 37 °C for 3 h. The supernatant was then removed, and 100 μL DMSO was added to the cell pellet, which was mixed and read at 570 nm.

After incubation with the APs for 1 h, the cells were treated with ox-LDL for 6 h. The cells were then harvested and washed with PBS. The production of Intracellular ROS was measured by incubating the cells with 10 μM 5-(and-6)-carboxy-2′, 7′-dichlorodihydrofluorescein diacetate (carboxy-H2 DCFDA; Invitrogen), a cell-permeable indicator for ROS generation, at 37 °C for 30 min, then the cells were washed with PBS. The ROS generation was determined based on the mean fluorescent intensity measured by flow cytometry (Beckman Coulter, Brea, CA, USA).

### 2.7. Quantitation of Gene Expression

The total RNA was isolated from the mouse livers or cultured cells using the TRIzol Reagent (Takara, TaKaRa Biotechnology, Dalian, China) according to the manufacturer’s protocol. The mRNA concentrations were measured using a Nano Drop instrument (Thermo Electron, Wilmington, NC, USA). The appropriate quantity of cDNA was generated with a PrimeScript RT reagent kit (TaKaRa, Tokyo, Japan) according to the manufacturer’s protocol. qRT-PCR was performed on the ABI 7500 Fast Real Time PCR system (Applied Biosystems, Foster City, CA, USA) using SYBR Green detection chemistry (TaKaRa, Tokyo, Japan). All samples were measured in triplicate and the mean value used for the comparative analysis. Quantitative measurements were calculated using the 2^−∆∆Ct^ method. GAPDH served as the housekeeping gene for the comparisons of the gene expression data. The primer sequences for the qRT-PCR analyses of the genes were selected according previous studies and are listed in Table 1.

**Table 1:** Primer sequences (from 5′ to 3′) for measuring mRNAs using real-time PCR.

mRNA	Forward Primer	Reverse Primer
rat ICAM-1	AGATCATACGGGTTTGGGCTTC	TATGACTCGTGAAAGAAATCAGCTC
rat VCAM-1	TGTGGAAGTGT GCCCGAAAT	TGCCTTGCGGATGGTGTAC
rat CCL2	ATGCAGGTCTCTGTCACGCT	GGTGCTGAAGTCCTTAGGGT
rat GAPDH	CATGTTCGTCATGGGTGTGAACCA	ATGGCATGGACTGTGGTCATGAGT
mouse PPARα	TCAGGGTACCACTACGGAGTTCA	CCGAATAGTTCGCCGAAAGA
mouse SCAP	ACTGGACTGAAGGCAGGTCAA	GCCTCTAGTCTAGGTCCAAAGAGTTG
mouse SCD-1	CAGTGCCGCGCATCTCT	CCCGGGATTGAATGTTCTTG
mouse HMG-CoA R	CCCAGTTGTGCGTCTTCCA	TTCGAGCCAGGCTTTCACTT
mouse HMG-CoA S	GCCGTGAACTGGGTCGAA	GCATATAGCAATGTCCTGCAA
mouse Nrf2	GGCCCAGCATATCCAGACA	TGTGGGCAACCTGGGAGTAG
mouse GCLm	ACATTGAAGCCCAGGATTGG	CTCTTCACGATGACCGAGTACCT
mouse SOD-1	A GCCCGGCGGATGAAG	CCTTTCCAGCAGTCACATTGC
mouse GAPDH	ATGTTTGTGATGGGTGTGAACCAG	TAGCCATATTCATTGTCATACCAGG

### 2.8. Enzyme-Linked Immunosorbent Assay

The concentrations of ICAM-1, VCAM-1 and CCL2 in plasma, as well as the supernatants collected from treated RAECs were determined using rat enzyme-linked immunosorbent assay kits (Boster, Wuhan, China) according to the instructions provided by manufacturer. The color absorbance at 450 nm was measured using a Bio-Rad microplate reader.

### 2.9. Western Blotting

After treatments, the cells were washed with cold PBS and lysed in 100 μL RIPA buffer containing 10 mM PMSF (Beyotime, Haimen, China). The nuclear extracts were prepared using a nuclear extraction kit (Sangon Biotech, Shanghai, China) according to the instructions provided by manufacturer. The protein concentrations were evaluated using a BCA Protein Assay Kit (Cwbiotech, Beijing, China). After boiling at 100 °C for 5 min, equal amounts of total protein were separated by SDS/PAGE (10% gel) and transferred to nitrocellulose membranes. The membranes were incubated in 5% fat-free milk powder in Tris-buffered saline solution-Tween-20 (TBST) for 1 h at room temperature and then exposed to the primary antibodies at 4 °C overnight. After rinsing in TBST, the membranes were incubated with the secondary antibodies for 1 h at room temperature. Antibodies specific for p-Erk1/2, Erk1/2, p-p38, p38, IκBα, NF-κΒ (p65), and β-actin were purchased from Cell Signaling Technology. The immunoreactive bands were visualized by a two-color infrared imaging system (Odyssey; LI-COR, Lincoln, NE, USA).

### 2.10. Statistical Analysis

The data are presented as the means ± S.E.M. The statistical analyses were conducted using two-way analysis of variance (ANOVA) or Student’s *t* test with SPSS software (version 16.0; SPSS, Chicago, IL, USA). A value of *p* < 0.05 indicated significance.

## 3. Results

### 3.1. APs Prevent Atherosclerotic Plaque Development

**Figure 1:**
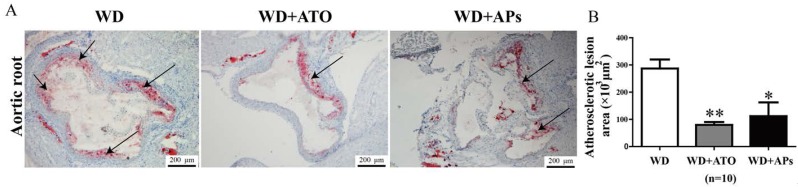
Quantification of atherosclerotic lesions in the aortic sinuses of ApoE^−/−^ mice fed with a Western-type diet (WD). **A**: Representative images showing the atherosclerotic lesions in the aortic sinuses of ApoE^−/−^ mice fed with WD, WD + atorvastatin (ATO) or WD + apple polyphenols (APs). The frozen aortic roots were stained with Oil Red O and counterstained with hematoxylin; **B**: Quantitative analysis of atherosclerotic lesion areas. The values are presented as the means ± S.E.M., *n* = 10 per group. * *p* < 0.05, ** *p* < 0.01 *vs.* WD mice (control group).

We examined the effects of APs on the lesion formation in atherosclerotic conditions using ApoE^−/−^ mice. The results from the Oil Red O-stained aortic root lesions revealed that the treatment with APs or atorvastatin for 12 weeks significantly reduced the lesion size (Figure 1A) by 61.02% ± 19.39% (*p* < 0.05) or 72.22% ± 9.07% (*p* < 0.01) , respectively, compared to the WD mice, respectively (Figure 1B). There was not a significant difference between APs and atorvastatin groups.

### 3.2. APs Normalize the Body Weight and Metabolic Parameters in the Apo ^−/−^ Mice Fed with the Western-Type Diet

**Figure 2:**
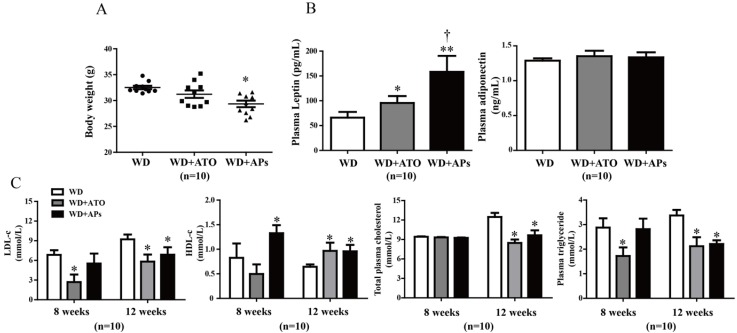
The APs treatment normalized the up-regulated body weight and metabolic parameters in the WD-fed ApoE ^−/−^ mice. **A**: Assessment of body weight for the weeks 12; **B**: Concentrations of leptin and adiponectin in plasma; **C**: Total cholesterol, triglycerides, LDL-c, and HDL-c were quantified by enzymatic assays. The values are presented as the means ± S.E.M., *n* = 10 per group. * *p* < 0.05, ** *p* < 0.01, *** *p* < 0.001 *vs.* WD mice; † *p* < 0.05 *vs.* WD + ATO mice.

Treatment with the APs markedly normalized body weight that was upregulated by 12 weeks of WD feeding in ApoE^−/−^ mice (Figure 2A). To investigate the anti-hyperlipidemic effects of APs administration, we also measured the plasma leptin and adiponectin levels. We found that the concentration of leptin was increased by 139% in the plasma of the ApoE^−/−^ mice fed the WD, whereas there were no significant differences among all groups in the plasma adiponectin levels among the groups (Figure 2B). In addition, treatment with APs led to a significant decreases in the total cholesterol and triglyceride s in the plasma, compared to control group, accompanied by a 48.75% increases in the circulating HDL-c, and a 29.08% decreases in the circulating LDL-c level (Figure 2C). However, neither APs nor ATO treatment could completely return the animals to baseline. Total cholesterol and triglycerides in the plasma of the WD-fed mice, even with either treatment, were still much higher than those in the ApoE^−/−^ mice fed on a standard chow diet (Figure S1).

### 3.3. APs Ameliorate Liver Lipogenesis and Hepatic Steatosis in WD-fed ApoE^−/−^ Mice

**Figure 3:**
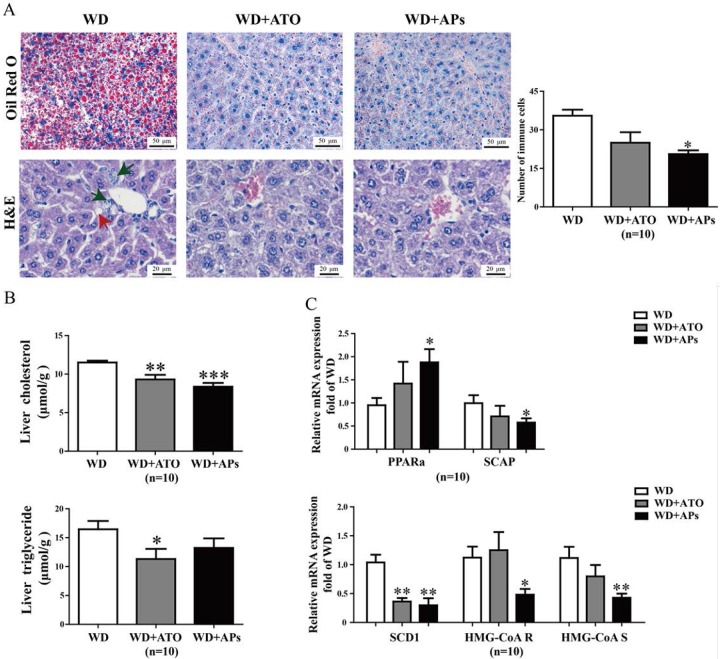
The APs attenuate hepatic lipid deposition, lipogenesis and inflammatory cell infiltration in the livers of WD-fed ApoE^−/−^ mice. **A**: Hepatic lipid deposition was measured in WD-fed ApoE^−/−^ mice treated with APs (100 mg/kg/day) or ATO (10 mg/kg/day) for 12 weeks. Microscopic examination of Oil Red O and H&E stained-liver sections revealed decreased accumulation of neutral fat and steatosis in the APs- or ATO-treated mice compared to the WD model mice. The red arrow shows the steatosis in the liver, and the black arrow shows the infiltration of inflammatory cells; **B**: Hepatic TG and total cholesterol levels were measured in the ApoE^−/−^ mice fed with WD, WD + ATO, or WD + Aps; **C**: mRNA levels of the hepatic genes—*SCAP*, *PPARα*, *SCD-1*, *HMG-CoA R* and *HMG-CoA S* were determined by real-time PCR. These genes are involved in lipid metabolism. The values are presented as the means ± S.E.M., *n* = 10 per group. * *p* < 0.05, ** *p* < 0.01, *** *p* < 0.001 *vs.* WD mice.

WD leads to an impaired balance between hepatocyte lipid uptake and lipogenesis, resulting in lipid deposition in the liver. To investigate the effects of the APs on WD-induced hepatic steatosis, we examined the hepatic triglyceride and cholesterol accumulation. Microscopic examination of the Oil Red O-stained tissues showed that fat droplets, an important histological feature for the diagnosis of human nonalcoholic steatohepatitis (NASH), appeared after 12 weeks of WD feeding in the control group. The H&E-stained liver sections showed slight inflammatory cell infiltrations within the microvesicular steatoses and veins. Treatment with the APs or atorvastatin significantly normalized the histological severity of steatohepatitis (Figure 3A). Consistent with the hepatic histological results, the biochemical analysis revealed the inhibitory effects of the APs on cholesterol deposition. The total cholesterol concentration in the AP-treated group was markedly lower than that in control group, while no significant difference was observed for the triglyceride level (Figure 3B). We also observed suppression of the transcription of hepatic lipogenic gene in s in the AP-treated mice. The transcription of the peroxisome protein, *PPARα* in the AP-treated mice increased by 90.2% (*p* < 0.05) relative to the control mice, while the transcriptions of *SCAP* was suppressed by 42.05% (*p* < 0.04). In addition, the expression of the mRNA for *SCD-1*, *HMG-CoA R*, and *HMG-CoA S* mRNA was reduced by 71.34% (*p* < 0.03), 57.22%, and 62.13% (*p* < 0.005) in the AP-treated mice (Figure 3C).

### 3.4. APs Prevent the Liver from Hepatic Oxidative Stress

**Figure 4:**
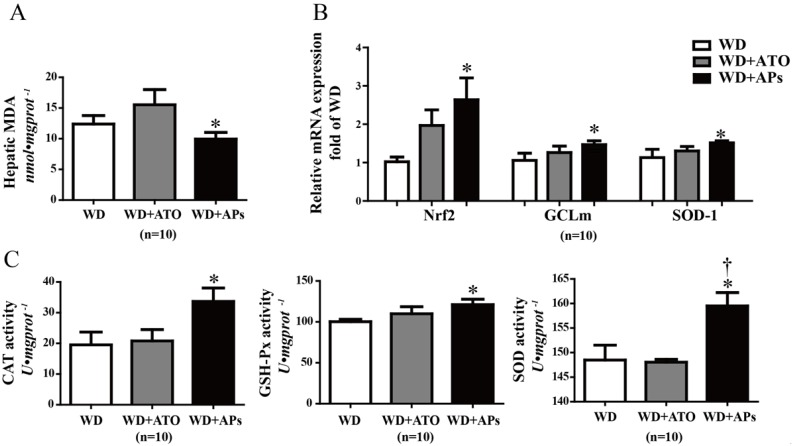
The APs attenuate hepatic oxidative stress in the WD-fed ApoE^−/−^ mice. **A**: The hepatic concentration of MDA was measured by monitoring the formation of thiobarbituric acid-reactive substances; **B**: Expression of the antioxidant gene *Nrf2*, *GCLm* and *SOD-1* in the liver tissues of ApoE^−/−^ mice fed with the WD or WD+APs (100 mg/kg/day) or WD+ATO (10 mg/kg/day) for 12 weeks; **C**: hepatic CAT, GSH-Px and superoxide dismutase (SOD) activities were measured in the liver tissues. The values are presented as the means ± S.E.M., *n* = 10 per group. * *p* < 0.05, ** *p* < 0.01, *** *p* < 0.001 *vs.* WD mice; † *p* < 0.05 *vs.* WD + ATO mice.

APs are best known for their antioxidant properties. We found that supplementation with these extracts significantly lowered the MDA levels in the livers of the WD-fed mice, which suggested that they have a protective effect against oxidative stress (Figure 4A). To evaluate the underlying mechanism by which the APs participate hepatic endogenous antioxidant defense, qRT-PCR analysis was performed to measure the expression of the genes involved in the hepatic oxidative stress. The APs significantly increased the transcription levels of Nrf2 gene as well as its targeting genes including GCLm and SOD-1 (Figure 4B). Furthermore, the activities of CAT, GSH-Px, and SOD were significantly increased by the APs treatment (Figure 4C).

### 3.5. APs Attenuate CD68 Expression in the Aortic Root, and CCL2 and VCAM-1 Levels in Plasma

**Figure 5:**
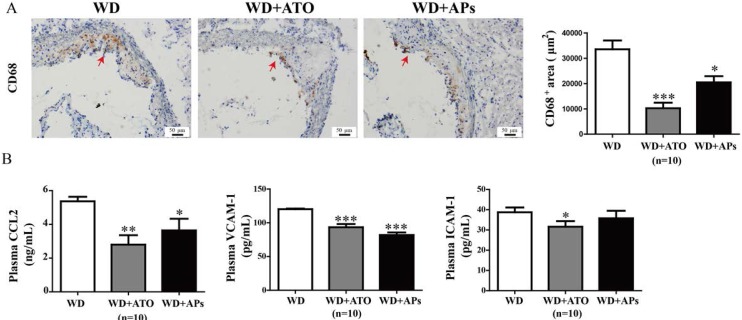
Treatment with APs or ATO caused a decrease in macrophage positive areas in the atherosclerotic lesions, as well as lower plasma levels of CCL2, VCAM-1, and ICAM-1 in the WD-fed ApoE^−/−^ mice. **A**: Immunohistochemical visualization and semi-quantitative analysis of CD68-positive macrophages (brown colored areas) in the atherosclerotic lesions; **B**: The concentrations of CCL2, ICAM-1 and VCAM-1 protein in plasma were measured by ELISA. The values are presented as the means ± S.E.M., *n* = 10 per group. * *p* < 0.05, ** *p* < 0.01, *** *p* < 0.001 *vs.* WD mice.

Immunoreactivity for CD68 showed an accumulation of macrophages in the lipid cores and in the shoulders of the atherosclerotic plaques. Our results showed that the macrophage infiltration into the plaque was reduced by 39% and 69% in WD + APs mice and WD + ATO mice, respectively (Figure 5).

The upregulation of levels of the circulating CCL2, VCAM-1, and ICAM-1 protein in atherosclerotic mice indicated the inflammatory responses and endothelial injury in ApoE^−/−^ mice fed with the WD. The ApoE^−/−^ mice treated with APs or atorvastatin treatment showed markedly decreased levels of CCL2 and VCAM-1 level compared with the model mice (Figure 5), whereas atorvastatin but not the APs significantly lowered the plasma ICAM-1 levels.

### 3.6. APs Suppress ox-LDL-Induced Pro-Inflammatory Factors and Alleviates Oxidant Stress in ox-LDL-Treated Endothelial Cells

Vascular endothelial cells treated with ox-LDL were used as a model to evaluate the anti-inflammatory effects of the APs. We previously showed that ox-LDL (10–100 μg/mL) induces ICAM-1, CCL2, and E-selectin expression in a concentration-dependent manner in RAECs. Indeed, the increases in the expression of adhesion molecules following LDL-stimulation have been reported to promote the adhesion of human monocytes to cultured endothelial cells [21]. Here, we isolated RAECs from the aortas of healthy adult rats. We confirmed that these cultures consisted of more than 90% endothelial cells with endothelial marker factor VIII staining (data not shown). In addition, we observed no decline in the cell viability following a 24 h incubation with the APs alone (data not shown), which suggests limited toxicity of the AP treatment to the RAECs at the working concentrations.

**Figure 6:**
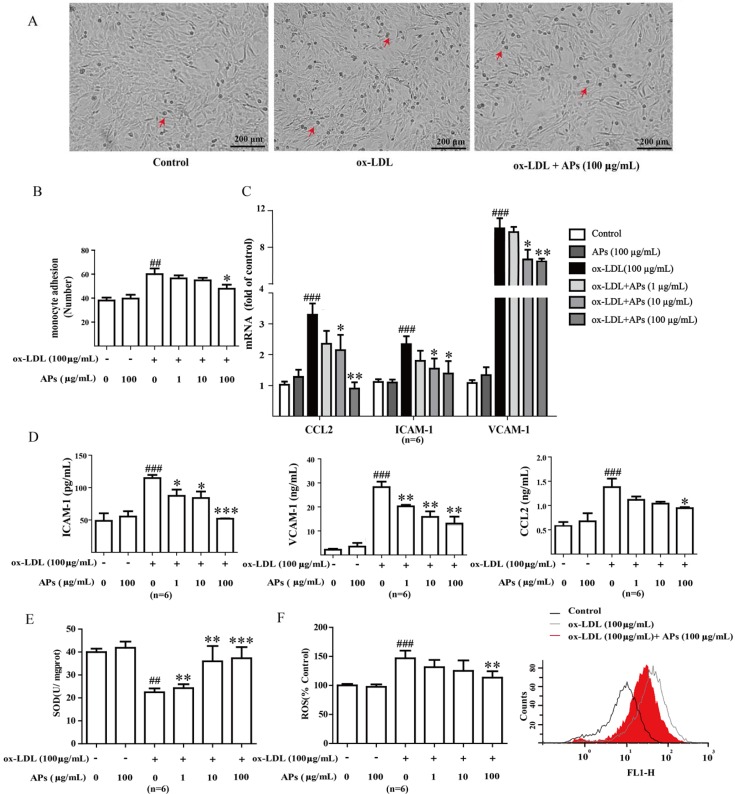
APs attenuate adhesion, decrease inflammatory factors and ameliorate oxidant stress in RAECs. **A**: Representative images of ox-LDL-induced monocyte adhesion to RAECs; **B**: APs treatment inhibits the ox-LDL-induced monocyte adhesion to RAECs in a concentration-dependent manner; **C**: CCL2, ICAM-1, and VCAM-1 gene expression in RAECs; **D**: CCL2, ICAM-1, and VCAM-1 level in RAECs supernatant; **E**: Activity of SOD in RAECs; **F**: Representative FACS plot of intracellular reactive oxygen species (ROS) and production in RAECs. The values are presented as the means ± S.E.M., *n* = 6 per group. # *p* < 0.05, ## *p* < 0.01, ### *p* < 0.001 *vs.* control group (0 μg/mL). * *p* < 0.05, ** *p* < 0.01, *** *p* < 0.001 *vs.* ox-LDL alone.

We showed that the incubation of the vascular endothelial cell with ox-LDL led to endothelial dysfunction and monocyte adhesion (Figure 6A). Pretreatment with the APs for 1 h prevented the ox-LDL-induced monocyte froms from adhering to the RAECs in a concentration-dependent manner (Figure 6B). Given that the AP treatment caused a robust inhibition of the monocyte adhesion and the production of pro-inflammatory cytokines in mice, we hypothesized that the APs treatment would also be able to suppress chemokine expression and the surface molecules in the endothelial cells. Exposing the RAECs to ox-LDLs significantly increased the mRNA transcription (Figure 6C) and protein secretion (Figure 6D) of ICAM-1, VCAM-1, and CCL2. The APs treatment significantly normalized such the ox-LDLs-induced expression of adhesion molecules and chemokines in the RAECs (Figure 6C,D).

Moreover, a 6 h incubation with ox-LDL diminished the SOD activity in the RAECs, which was accompanied by a significantly increase in ROS production. Treatment with the APs normalized the SOD and ROS level in a concentration-dependent manner (Figure 6E,F).

### 3.7. APs inhibit the ox-LDL-Induced MAPK and NF-κB Pathway Activation in Endothelial Cells

**Figure 7:**
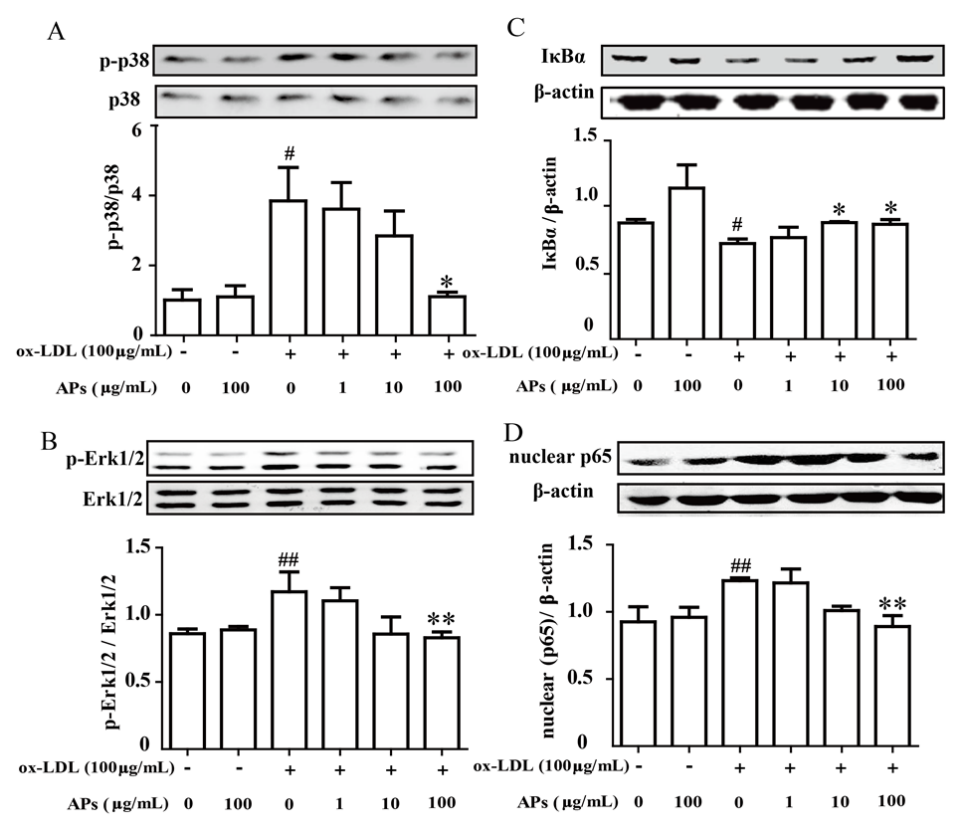
APs inhibit the MAPK and NF-κB activation induced by ox-LDL in RAECs. RAECs were cultured in media with various concentrations of APs for 1 h, followed stimulation with ox-LDL for 30 min to assess the MAP kinase pathway or for 60 min to assess the NF-κB pathway. **A** and **B**: Activation of p38 or Erk1/2 was determined by immunoblotting using phosphor-specific (p-) antibodies. Representative blots from three independent experiments are shown. The quantitation of the ratio between phosphorylated protein/total proteins in the cell lysates is shown in the bar graphs; **C** and **D**: The nuclear and cytosolic fractions of NF-κB were extracted and then assessed by Western blot. The results are shown as the means ± S.E.M. # *p* < 0.05, ## *p* < 0.01 *vs.* control group. * *p* < 0.05, ** *p* < 0.01 *vs.* ox-LDL alone.

Because the inhibitory effects of the APs on the production of pro-inflammatory cytokines were observed both *in vivo* and *in vitro*, we further investigated the intracellular signaling pathways involved in this effect. Incubation of the RAECs with ox-LDL induced a significant increase in phospho-Erk MAP kinase and p38 MAP kinase—the upstream targets for NF-κB activation. However, a 1 h pretreatment with the APs significantly suppressed the 30 min ox-LDL-induced phospho-Erk and phospho-p38 MAP kinase that were induced by a 30 min treatment with ox-LDL in the RACEs (Figure 7A,B). Moreover, the APs also suppressed the ox-LDL-induced activation of the NF-κB signaling pathway in the RAECs. This treatment showed a dose-dependent inhibition of the ox-LDL-induced degradation of IκBα in the cytosolic fraction, as well as an amelioration of the ox-LDL-induced translocation of p65 subunit from the cytoplasm into the nucleus (Figure 7C,D). These results indicate that the APs treatment can effectively attenuate the ox-LDL-activated MAPK/NF-κB axis, which further results in a decrease in the expression and production of pro-inflammatory genes by the RAECs.

## 4. Discussion

The adverse events associated with cardiovascular disease are found to be correlated with nonalcoholic steatohepatitis, include obesity, dyslipidemia, and oxidative stress, which are independent of metabolic abnormalities. A dietary supplements made from APs may be a novel antioxidant therapy to decrease the risk of chronic diseases, such as diabetes and cancer [5,6]. Our studies demonstrated a beneficial effect of APs in preventing plaque formation in ApoE^−/−^ WD-fed mice by regulating the expression of genes that are involved in lipid metabolism and antioxidant defense. The potency of the APs treatment in reducing atherosclerosis was not greater than that of atorvastatin. However, there is still a strong potential for the application of AP treatment because this type of supplement can be consumed as a food ingredient with higher liver safety. More importantly, we have demonstrated that the decreased endothelial lesion formation in the WD-fed ApoE^−/−^ mice could be attributed to the anti-inflammatory and anti-oxidative mechanisms of the APs, which is involved inhibition of the endothelial MAPK and NF-κB pathways to suppress the expression of the pro-inflammatory cytokines.

The progression of atherosclerosis from early development to plaque rupture begins with the adhesion of leukocytes to vascular endothelium, followed by loading with lipid; these cells eventually mature in the intima [22]. We observed that 12-week treatment with the APs remarkably reduced the lesion area in the aortic sinuses by 61.02% ± 19.39% in the ApoE^−/−^ mice fed with WD. This reduction of plaque formation was consistent with a previous study where ApoE^−/−^ mice were fed with a normal chow diet [23]. Daily administration of APs for 12 weeks also lowered the body weight and plasma lipid levels of the WD-fed ApoE^−/−^ mice. Our results for the plasma metabolic parameters indicated that the APs had anti-atherogenic and anti-obesity effects of APs, which were related to the decreased plasma TC and TG levels. Moreover, in this study, the APs treatment elevated the plasma levels of leptin, which plays an important role in the regulation of body weight and energy balance. It has also been reported that deficiency of either leptin or its receptor results in marked increases in both plasma cholesterol and triglyceride concentrations and increased lesion formation. Leptin-deficient mice have the potential for hypertriglyceridemia and hypercholesterolemia [24].

In the livers of the APs-treated mice, we observed that the transcription of hepatic lipogenic enzymes was lower than those of the WD-fed mice. The APs resulted in significantly lower levels of total cholesterol, low-density lipoprotein cholesterol, hepatic lipid content, and lipid droplet numbers and size. The APs also suppressed the WD-induced increase in expression of lipogenic genes including *SCD-1*. The expression of cholesterol metabolism-related genes [25], such as *HMG-CoA R* and *HMG-CoA S*, were also down-regulated in the WD-fed mice. In contrast, the APs treatment-enhanced genes expression of *PPARα* may upregulate fatty acid β-oxidation in the liver [26]. The APs may prevent the WD-induced fat accumulation by regulating lipid metabolism-associated gene expression, and may be a useful natural product for the prevention of nonalcoholic fatty liver disease.

In addition, our results showed that the APs increased the antioxidant enzyme activity in the livers. Free radicals or ROS are oxidants that can damage cellular lipids, proteins, and DNA, leading to oxidative stress and inducing liver injury. Current research suggests that CAT, GSH-Px and SOD are the first line of defense against oxidative injury, preventing hepatic lipid metabolism from being disrupted by free radicals or ROS [27,28]. In the present study, long-term intake of the APs enhanced the CAT, GSH-Px and SOD activities, possibly through the up-regulation of their transcription via the Nrf2 signaling pathway, which was up-regulated by APs. Nrf2 has a crucial role in protecting the hepatic cells that could lead to the development of liver diseases [29]. In the WD-fed mice, the antioxidant enzymes were up-regulated by the APs treatment, which may have strengthened the endogenous defense against oxidative stress in the liver. Consistently, hepatic MDA was also significantly decreased in the same mice [30].

A high-fat diet induces an increase in small dense LDL-c and a decrease in HDL-c, which are highly susceptible to oxidation [31]. This pro-inflammatory mediator can damage vascular endothelial cell, recruit leukocytes to the endothelial cells, and induce proliferation of the smooth muscle cells. A long-term high-fat diet increases steatohepatitis due to the generation of ROS and impairs the antioxidant defense mechanisms in the liver [32]. Here, we showed that treatment with the APs led to a significant reduction in the level of LDL-c and an increase in the level of HDL-c in the plasma, which provides new evidence to support the potential of using APs to prevent vascular inflammatory responses and hepatic steatosis.

Atherosclerosis is characterized by foam cell formation and accumulation in the subendothelial spaces of the arterial vessels [33,34]. Foam cells are mainly derived from CD68-positive monocytes and macrophages that migrate from the vascular lumen to the wall [35]. The agents that regulate CD68 expression and macrophage infiltration may determine in the progression or regression of atherosclerosis [36]. In the present study, we found that the APs and ATO reduced the numbers of CD68-positive cells by 39% and 69%, respectively (Figure 5). This result suggested that the APs may inhibit macrophage infiltration in the plaque.

The leukocyte recruitment cascade is a sequence of adhesion and activation events that requires the participation of adhesion molecules and chemokines. CCL2 (MCP-1) participates in the pathogenesis of atherosclerotic lesion formation by promoting the directed migration of leukocytes or macrophages [37]. ICAM-1 and VCAM-1 induce firm adhesion of the inflammatory cells at the vascular surface. The regulation of the production of adhesion molecules and chemokines production has been proposed as a potential target for new therapeutics in the prevention of atherosclerosis [38]. We observed that the anti-inflammatory and anti-adhesion effects of the APs on the endothelium are preserved both *in vivo* and *in vitro*. Our data showed that APs significantly suppressed the ox-LDL-induced expression of CCL2, ICAM-1, and VCAM-1 in the RAECs. These effects may contribute to the reduced monocyte adhesion to endothelium.

A complex array of intracellular signaling pathways, including mitogen-activated protein kinases (MAPKs) and nuclear transcriptional factors, are involved in the regulation of chemokines and adhesion molecule expression [39]. NF-κB is proposed to be an integrator of many processes that affects the formation of atherosclerotic lesions. As a family of transcriptional factors, the NF-κB pathway regulates the transduction of many inflammatory factors that participate in atherosclerotic plaques formation. Activation of NF-κB requires the phosphorylation and ubiquitination of IκBα followed by its proteolytic degradation by a proteasome complex [40]. In the present study, we found that ox-LDL induced higher levels of NF-κB phosphorylation and degradation of IκBα in RAECs, and that these processes could be normalized by pretreatment with the APs. The decreases in activated endothelial cells may be an important mechanism by which APs could regulate the production of CCL2, ICAM-1, and VCAM-1.

In addition, we confirmed that the APs treatment suppressed the ox-LDL-induced phosphorylation in the p38 MAPK and Erk1/2 pathway in RAECs. These pathways have been identified as major elements up-stream of the NF-κB pathway and as being essential for the ox-LDL-induced and NF-κB-dependent gene expression. In our results, the upregulated VCAM-1 and ICAM-1 could be attributed to the activation of the p38 signaling pathway, in other studies, a p38 inhibitor SB203580, attenuated the expression of VCAM-1 and ICAM-1, with or without ox-LDL stimulation [41,42].

## 5. Conclusions

In summary, we observed for the first time that a pre-supplementation with APs can prevented the ox-LDL-induced MAPK/NF-κB activation and reduce the subsequent endothelial inflammation, which are early critical steps in the formation of arteriosclerotic lesions. We observed that a 12 weeks treatment with APs significantly reduced the plaque size in the aortic sinus of WD-fed ApoE^−/−^ mice. We also demonstrated that treatment with the APs effectively attenuated hepatic steatosis by ameliorating oxidative stress and lipogenesis in the liver. Taken together, our data suggest beneficial effects of APs on plaque formation, which are mediated by their anti-inflammatory properties. Given the benefits of APs on lipid modulation and antioxidant defense, a primary approach based on these effects of APs would contribute to the prevention of atherosclerosis.

## Supplementary Files

Supplementary File 1

## Abbreviations

ApoE*^−/−^* mice: Apolipoprotein E knockout mice
ATO: atorvastatin
CAT: catalase
CCL-2: The chemokine (C-C motif) ligand 2
CVD: Cardio vascular disorder
DAPI: 4′,6-diamidino-2-phenylindole
Erk: extracellular regulated protein kinases
Fe/Asc: iron-ascorbate
GCLm: glutamate-cysteine ligase modifier subunit
GPx: glutathione peroxidase
GPX-1: GSH peroxidase
GSH-Px: glutathione peroxidase
HDL: high-density lipoprotein
HDL-c: high-density lipoprotein cholesterol
H&E: hematoxylin and eosin
HMG-CoA R: HMG-CoA reductase
HMG-CoA S: HMG-CoA Synthase
ICAM-1: intracellular adhesion molecule-1
IκB-α: nuclear factor of kappa-light-polypeptide gene enhancer in B-cells inhibitor, alpha
IL-1β: Interleukine-1 beta
LDL-c: high-density lipoprotein cholesterol
LPS: lipopolysaccharide
MAPKs: mitogen-activated protein kinases
MDA: malondialdehyde
MTT: 3-(4,5-dimethyl-2-thiazolyl)-2,5-diphenyl-2-H-tetrazolium bromide
NASH: nonalcoholic steatohepatitis
NF-κB: nuclear factor kappa B
Nrf2: nuclear factor erythroid-2
ox-LDL: oxidized low-density lipoprotein
PPARα: peroxisome proliferator-activated receptor-α
RAECs: rat aortic endothelial cells
ROS: reactive oxygen species
RPBMNCs: rat peripheral blood mononuclear cells
SCAP: SREBP cleavage-activating protein
SCD-1: stearoyl-CoA desaturase-1
SOD: superoxide dismutase
SREBP-2: sterol regulatory element-binding protein-1c
TNF-α: tumor necrosis factor-a
VCAM-1: vascular adhesion molecule-1
WD: Western-type diet

## References

[1] Koutsos A., Lovegrove J.A., Tuohy K., del Rio D. (2015). An apple a day keeps the doctor away: Inter-relationship between apple consumption, the gut microbiota and cardiometabolic disease risk reduction. Diet-Microbe Interactions in the Gut: Effects on Human Health and Disease.

[2] Lotito S.B., Frei B. (2004). Relevance of apple polyphenols as antioxidants in human plasma: Contrasting *in vitro* and *in vivo* effects. Free Radic. Biol. Med..

[3] Dauchet L., Amouyel P., Dallongeville J. (2009). Fruits, vegetables and coronary heart disease. Nat. Rev. Cardiol..

[4] Corcoran M.P., McKay D.L., Blumberg J.B. (2012). Flavonoid basics: Chemistry, sources, mechanisms of action, and safety. J. Nutr. Gerontol. Geriatr..

[5] Boyer J., Liu R.H. (2004). Apple phytochemicals and their health benefits. Nutr. J..

[6] Knekt P., Kumpulainen J., Jarvinen R., Rissanen H., Heliovaara M., Reunanen A., Hakulinen T., Aromaa A. (2002). Flavonoid intake and risk of chronic diseases. Am. J. Clin. Nutr..

[7] Shoji T., Akazome Y., Kanda T., Ikeda M. (2004). The toxicology and safety of apple polyphenol extract. Food Chem. Toxicol..

[8] Ammirati E., Moroni F., Norata G.D., Magnoni M., Camici P.G. (2015). Markers of inflammation associated with plaque progression and instability in patients with carotid atherosclerosis. Mediators. Inflamm..

[9] Khan R., Spagnoli V., Tardif J.C., L’Allier P.L. (2015). Novel anti-inflammatory therapies for the treatment of atherosclerosis. Atherosclerosis.

[10] Sahebkar A., Chew G.T., Watts G.F. (2014). New peroxisome proliferator-activated receptor agonists: Potential treatments for atherogenic dyslipidemia and non-alcoholic fatty liver disease. Expert Opin. Pharmacother..

[11] Tiong A.Y., Brieger D. (2005). Inflammation and coronary artery disease. Am. Heart J..

[12] Kim E.J., Kim B.H., Seo H.S., Lee Y.J., Kim H.H., Son H.H., Choi M.H. (2014). Cholesterol-induced non-alcoholic fatty liver disease and atherosclerosis aggravated by systemic inflammation. PLoS ONE.

[13] Tabas I., Williams K.J., Borén J. (2007). Subendothelial lipoprotein retention as the initiating process in atherosclerosis: Update and therapeutic implications. Circulation.

[14] Ashraf M.Z., Kar N.S., Podrez E.A. (2009). Oxidized phospholipids: Biomarker for cardiovascular diseases. Int. J. Biochem. Cell Biol..

[15] Wouters K., van Gorp P.J., Bieghs V., Gijbels M.J., Duimel H., Lütjohann D., Kerksiek A., van Kruchten R., Maeda N., Staels B. (2008). Dietary cholesterol, rather than liver steatosis, leads to hepatic inflammation in hyperlipidemic mouse models of nonalcoholic steatohepatitis. Hepatology.

[16] Hermida N., Balligand J.L. (2014). Low-density lipoprotein-cholesterol-induced endothelial dysfunction and oxidative stress: The role of statins. Antioxid. Redox Signal..

[17] Pober J.S., Sessa W.C. (2007). Evolving functions of endothelial cells in inflammation. Nat. Rev. Immunol..

[18] Stoll G., Bendszus M. (2006). Inflammation and atherosclerosis: Novel insights into plaque formation and destabilization. Stroke.

[19] Bobryshev Y.V. (2006). Monocyte recruitment and foam cell formation in atherosclerosis. Micron.

[20] Srivastava R.A. (2011). Evaluation of anti-atherosclerotic activities of ppar-alpha, ppar-gamma, and lxr agonists in hyperlipidemic atherosclerosis-susceptible f(1)b hamsters. Atherosclerosis.

[21] O’Byrne D., Devaraj S., Islam K.N., Collazo R., McDonald L., Grundy S., Jialal I. (2001). Low-density lipoprotein (LDL)-induced monocyte-endothelial cell adhesion, soluble cell adhesion molecules, and autoantibodies to oxidized-LDL in chronic renal failure patients on dialysis therapy. Metabolism.

[22] Libby P., Ridker P.M., Hansson G.K. (2011). Progress and challenges in translating the biology of atherosclerosis. Nature.

[23] Auclair S., Milenkovic D., Besson C., Chauvet S., Gueux E., Morand C., Mazur A., Scalbert A. (2009). Catechin reduces atherosclerotic lesion development in apo E-deficient mice: A transcriptomic study. Atherosclerosis.

[24] Chai S.B., Sun F., Nie X.L., Wang J. (2014). Leptin and coronary heart disease: A systematic review and meta-analysis. Atherosclerosis.

[25] Hampton R., Dimster-Denk D., Rine J. (1996). The biology of HMG-CoA reductase: The pros of contra-regulation. Trends. Biochem. Sci..

[26] Pawlak M., Lefebvre P., Staels B. (2015). Molecular mechanism of PPARα action and its impact on lipid metabolism, inflammation and fibrosis in non-alcoholic fatty liver disease. J. Hepatol..

[27] Steinbrenner H. (2013). Interference of selenium and selenoproteins with the insulin-regulated carbohydrate and lipid metabolism. Free Radic. Biol. Med..

[28] Polimeni L., del Ben M., Baratta F., Perri L., Albanese F., Pastori D., Violi F., Angelico F. (2015). Oxidative stress: New insights on the association of non-alcoholic fatty liver disease and atherosclerosis. World J. Hepatol..

[29] Tang W., Jiang Y.F., Ponnusamy M., Diallo M. (2014). Role of Nrf2 in chronic liver disease. World J. Gastroenterol..

[30] Pan Q.R., Ren Y.L., Zhu J.J., Hu Y.J., Zheng J.S., Fan H., Xu Y., Wang G., Liu W.X. (2014). Resveratrol increases nephrin and podocin expression and alleviates renal damage in rats fed a high-fat diet. Nutrients.

[31] Griffin B.A. (2014). Nonpharmacological approaches for reducing serum low-density lipoprotein cholesterol. Curr. Opin. Cardiol..

[32] Wang S., Moustaid-Moussa N., Chen L., Mo H., Shastri A., Su R., Bapat P., Kwun I., Shen C.L. (2014). Novel insights of dietary polyphenols and obesity. J. Nutr. Biochem..

[33] Stöger J.L., Gijbels M.J., van der Velden S., Manca M., van der Loos C.M., Biessen E.A., Daemen M.J., Lutgens E., de Winther M.P. (2012). Distribution of macrophage polarization markers in human atherosclerosis. Atherosclerosis.

[34] McLaren J.E., Michael D.R., Ashlin T.G., Ramji D.P. (2011). Cytokines, macrophage lipid metabolism and foam cells: Implications for cardiovascular disease therapy. Prog. Lipid Res..

[35] Figueroa A.L., Subramanian S.S., Cury R.C., Truong Q.A., Gardecki J.A., Tearney G.J., Hoffmann U., Brady T.J., Tawakol A. (2012). Distribution of inflammation within carotid atherosclerotic plaques with high-risk morphological features: A comparison between positron emission tomography activity, plaque morphology, and histopathology. Circ. Cardiovasc. Imaging.

[36] Li L., Wang Y., Xu Y., Chen L., Fang Q., Yan X. (2014). Atorvastatin inhibits CD68 expression in aortic root through a GRP78-involved pathway. Cardiovasc. Drugs Ther..

[37] Lin J., Kakkar V., Lu X. (2014). Impact of MCP-1 in atherosclerosis. Curr. Pharm. Des..

[38] Rolin J., Maghazachi A.A. (2014). Implications of chemokines, chemokine receptors, and inflammatory lipids in atherosclerosis. J. Leukoc. Biol..

[39] Hopkins P.N. (2013). Molecular biology of atherosclerosis. Physiol. Rev..

[40] Asare Y., Shagdarsuren E., Schmid J.A., Tilstam P.V., Grommes J., El Bounkari O., Schütz A.K., Weber C., de Winther M.P., Noels H. (2013). Endothelial CSN5 impairs NF-κB activation and monocyte adhesion to endothelial cells and is highly expressed in human atherosclerotic lesions. Thromb. Haemost..

[41] Jiang J.X., Zhang S.J., Xiong Y.K., Jia Y.L., Sun Y.H., Lin X.X., Shen H.J., Xie Q.M., Yan X.F. (2015). EETs Attenuate Ox-LDL-Induced LTB4 Production and Activity by Inhibiting p38 MAPK Phosphorylation and 5-LO/BLT1 Receptor Expression in Rat Pulmonary Arterial Endothelial Cells. PLoS ONE.

[42] Jiang J.X., Zhang S.J., Liu Y.N., Lin X.X., Sun Y.H., Shen H.J., Yan X.F., Xie Q.M. (2014). EETs alleviate ox-LDL-induced inflammation by inhibiting LOX-1 receptor expression in rat pulmonary arterial endothelial cells. Eur. J. Pharmacol..

