# Supplementary material for: Apple Polyphenols Decrease Atherosclerosis and Hepatic Steatosis in ApoE−/− Mice through the ROS/MAPK/NF-κB Pathway
Source: Nutrients. 2015 Aug 24;7(8):7085–105. doi: 10.3390/nu7085324 (PMC4555163; doi:10.3390/nu7085324)
Supplement: Supplementary File 1 [file nutrients-07-05324-s001.doc]

Supplementary Materials


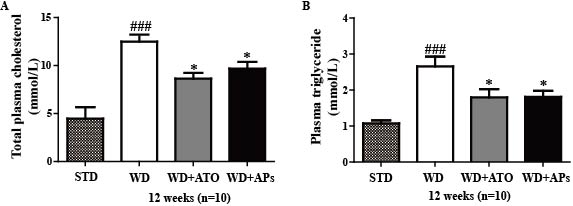


**Figure S1.** APs treatment normalizes plasma total cholesterol and triglycerides in ApoE^−/−^ mice fed with WD. **A**: Assessment of plasma total cholesterol in ApoE^−/−^ mice fed with STD, WD, WD + ATO, or WD + APs for 12 weeks. **B**: Concentration of triglycerides in plasma. Values are presented as mean ± S.E.M, *n* = 10 per group. * *p* < 0.05, ** *p* < 0.01, *** *p* < 0.001 *vs.* WD mice; ^#^ *p* < 0.05 *vs.* STD mice.
